# Biological and Socio-Cultural Factors Have the Potential to Influence the Health and Performance of Elite Female Athletes: A Cross Sectional Survey of 219 Elite Female Athletes in Aotearoa New Zealand

**DOI:** 10.3389/fspor.2021.601420

**Published:** 2021-02-18

**Authors:** Alison K. Heather, Holly Thorpe, Megan Ogilvie, Stacy T. Sims, Sarah Beable, Stella Milsom, Katherine L. Schofield, Lynne Coleman, Bruce Hamilton

**Affiliations:** ^1^Department of Physiology, School of Biomedical Sciences, University of Otago, Dunedin, New Zealand; ^2^WHISPA Group, High Performance Sport New Zealand, Auckland, New Zealand; ^3^School of Health, University of Waikato, Hamilton, New Zealand; ^4^Fertility Associates, Auckland, New Zealand; ^5^Auckland District Health Board, Auckland, New Zealand; ^6^Axis Sports Medicine, Auckland, New Zealand; ^7^High Performance Sport New Zealand, Auckland, New Zealand; ^8^Department of Obstetrics and Gynaecology, University of Auckland, Auckland, New Zealand; ^9^Sports Performance Research Institute New Zealand, Auckland University of Technology, Auckland, New Zealand

**Keywords:** elite athlete performance, hormonal contraceptive, oligomenorrhea, stress fracture, disordered eating and exercise behaviors, sociocultural factors

## Abstract

Health is a pre-requisite for optimal performance yet the parameters which govern health and performance of elite female athletes are little understood. The aim of this study was to quantify the health status of elite female athletes, and understand sociocultural factors influencing that status. The survey addressed demographic, health and athletic performance history, training load, contraceptive use, sport-specific appearance and performance pressures, and communication barriers. Three hundred and fifty-seven elite New Zealand female athletes were recruited to complete an on-line survey. Two hundred and nineteen athletes completed the survey. Oligomenorrhea/amenorrhea had been diagnosed in only 12% of athletes compared with 50% of athletes not on hormonal contraception who reported symptoms consistent with this diagnosis. Stress fractures and iron deficiency were common and associated with oligomenorrhoea/amenorrhea (*P* = 0.002), disordered eating (*P* = 0.009) or menorrhagia (*P* = 0.026). Athletes involved in individual sports (*P* = 0.047) and with higher training volumes (*P* < 0.001) were more likely to report a medical illness. Seventy-three percent of athletes felt pressured by their sport to alter their physical appearance to conform to gender ideals with 15% engaging in disordered eating practices. Barriers to communicating female health issues included male coaches and support staff, and lack of quality information pertaining to health. Elite female athletes may fail to reach peak performance due to specific health issues and undiagnosed pathology. Sociocultural factors influence the effectiveness of support of female's health and performance. Organizational and cultural change is required if elite female athletes are to combine optimal health with best performance.

## Introduction

Despite growth in female sport, data on the frequency and impact of health-related conditions in the elite female athlete remains limited (Mountjoy et al., 2014). Observational studies have highlighted the frequency of disordered eating, menstrual dysfunction, iron deficiency, and reduced bone mineral density in both elite and non-elite female athletes (Taylor and Rampton, 2015; Papageorgiou et al., 2018; McCall and Ackerman, 2019). Particularly in those sports requiring a low body mass or idealized appearance (Melin et al., 2019), these health issues may be underscored by an energy deficiency, recently characterized as the relative energy deficiency in sport syndrome (RED-S) (Logue et al., 2018). Energy deficiency may result in menstrual irregularity, estrogen deficiency, low bone mineral density and stress fractures (Mountjoy et al., 2014; McCall and Ackerman, 2019).

Socio-cultural factors also influence the health and performance of elite female athletes. For example, female athletes must navigate tensions between the social expectations of femininity, the physical requirements of sport-specific training, and the aesthetic expectations of specific sports (Krane et al., 2004). Furthermore, high-performance sporting cultures focusing on aesthetics, endurance, or weight, may normalize extreme dietary and training practices, potentially resulting in body dissatisfaction and disordered eating practices (Krane et al., 2004; de Bruin and Oudejans, 2018; Kantanista et al., 2018). Male coaches, social media, audiences and sponsors are recognized sources of pressure for female athletes (de Haan and Norman, 2019). Difficulty communicating with coaches and support staff who may be poorly informed on women's health issues, has also been identified as a specific challenge (Hines et al., 2019).

Despite more than 50% of the New Zealand team at the 2016 Olympics being female, few resources are available to National Sporting Organizations (NSO's) to specifically address female's health and performance considerations. Focusing on health and performance of elite female athletes, this study was designed to evaluate:

1. The prevalence of medical conditions, gynecological conditions or health related symptoms;
2. The presence of socio-cultural risk factors for negative health outcomes and;
3. The prevalence of hormonal contraceptive use.

## Methods

### Survey

Female athletes in elite and/or development programmes supported by High Performance Sport New Zealand (HPSNZ) were eligible to complete the survey. The online questionnaire was conceived and developed through a multi-disciplinary group (2 sociologists, 1 mechanistic physiologist, 1 applied physiologist, 2 endocrinologists, 2 sports physicians, 1 dietician, 1 nutrition scientist, 1 psychologist) established to facilitate healthy female athletes. An iterative approach was used to establish appropriate and relevant questions under the four main headings of demographics, health history, contraceptive history and sociological aspects of sport. The main construct was to unpack the prevailing medical conditions, attitudes, beliefs, and cultural considerations for elite NZ athletes. The final iteration, which took <20 min to complete, was tested on 4 athletes for language, comprehension and compliance with minor changes being made as a result of this evaluation (Supplementary Data).

### Procedure

A link to the survey via SurveyMonkey (SurveyMonkey Inc., San Mateo California, USA, www.surveymonkey.com) was emailed to all HPSNZ funded female athletes via their respective NSO's with two reminders at 6- and 10 weeks. Survey access closed after 3 months. Ethical approval was granted by the University of Waikato Human Research Ethics Committee [University of Waikato Human Research Ethics Committee (Health) 2018#52].

### Statistical Analysis

Multi-variant statistical analysis was performed by an independent statistician utilizing R version 3.6.0. Frequency tables were used to examine participant responses to *most* survey questions. Health conditions were examined in relation to demographic variables using chi-squared tests, or Fisher's exact tests in cases where cell counts were <5. For further exploration, sports were categorized as team or individual, and weightbearing or non-weightbearing. Diagnoses were categorized as injuries, illnesses or mental health issues.

## Results

**Figure 1:**
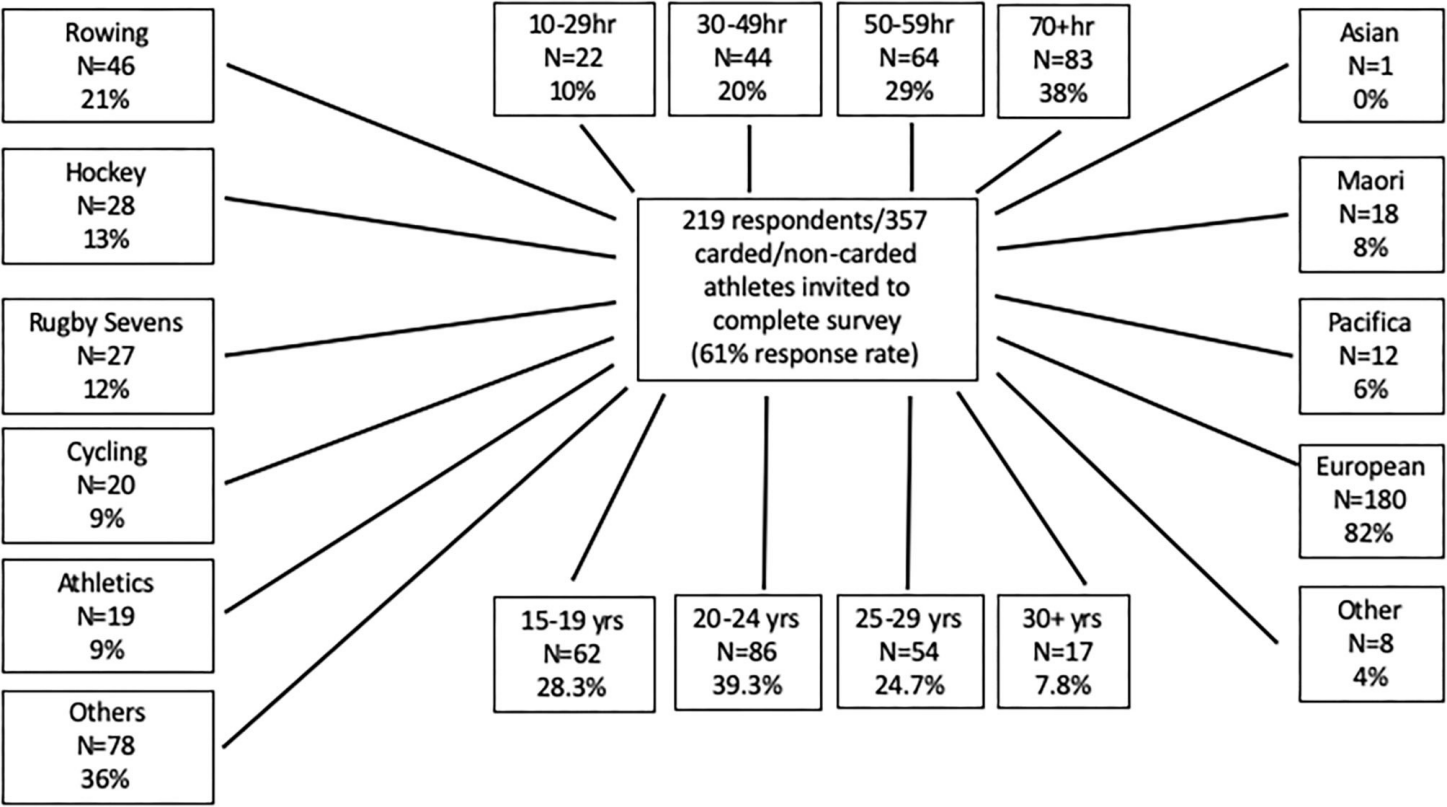
Flow chart showing athlete response rates based on questionnaire. The survey was sent to 357 elite athletes of which 219 responded. The athletes were divided into age groups 15–19, 20–24, 25–29, and 30+ years. Of the 219 athletes, most were European, with Maori, Pacifica, Asian, and others all represented. The athletes participated in a number of sports, and trained for 10–29, 30–49, 50–59 or 70+ h. Other sports include sailing (*n* = 18, 8%), netball (*n* = 15, 7%), canoe racing (*n* = 12, 6%), para sports (*n* = 7, 3%), triathlon (*n* = 6, 2%), swimming (*n* = 5, 2%), equestrian (*n* = 2, 1%), weightlifting (*n* = 1, 0%), canoe slalom (*n* = 1, 0%) other (*n* = 11) (only 218 athletes reported their sport).

Two hundred and nineteen responses were received from 357 invited athletes (61%). Respondents were 84%- elite and 16% development level athletes (Figure 1). Thirty-eight percent reported training at least 70 h per month, with training volumes higher in individual- compared with team sports (Supplementary Table 1, *p* < 0.001) (Figure 1). The majority of athletes (47%) had participated in elite level sports for 5–10 years with a further 25% for >10 years.

### General Medical History, Injuries, and Illness

The most prevalent clinically diagnosed injuries and illnesses include iron deficiency (47%, 99/212), stress fracture (23%, 50/219) and concussion (18%, 39/219) (Table 1). Further interrogation showed that injuries were more frequent in weight-bearing compared with non-weight bearing sports (*p* < 0.028, Supplementary Table 1) and in development vs. elite senior athletes (*p* = 0.002, Supplementary Table 1). A non-significant trend was observed in injury risk in team- vs. individual sports (*p* < 0.059, NS, Supplementary Table 1). Compared to team sports, it was more common for individual sport athletes to report a medically diagnosed illness (*p* = 0.047, Supplementary Table 1). Individual sport athletes reported higher training loads than team sport athletes (*p* < 0.001, Supplementary Table 1).

**Table 1:** Prevalence of clinically diagnosed injuries and illnesses.

**Diagnosed injury or illness**	***n***	**Respondents**	**Percent**
**Illness**			
Iron deficiency	99	212	47
Asthma	33	219	15
Depression/anxiety	25	219	11
Disordered eating	8	219	4
Hypothyroidism	3	219	1
Haemochromatosis	1	219	1
Oligo/amenorrhea	26	219	12
REDS/FAT	17	194	9
Endometriosis	16	194	8
Low bone density	5	219	2
Polycystic ovary syndrome	9	194	5
Sexually transmitted infection	13	194	7
Other gynecological diagnosis	6	194	3
**Injury**			
Stress fracture	50	219	23
Concussion	39	219	18
ACL rupture	14	219	6

Forty-six percent of respondents regularly used prescribed medications (98/212, Table 2), with the most common being some form of hormonal contraception (37%, 77/207, Table 3), non-steroidal anti-inflammatories (6%, 12/212, Table 2), asthma medications (6%, 12/212, Table 2) and anti-depressants (3%, 6/212, Table 2). Sixty-six percent of respondents (139/210) regularly used nutritional supplements, with the most common being protein, vitamins, caffeine, probiotics, and iron (Table 2). Medications (*p* = 0.036, Supplementary Table 1) and supplements (*p* = 0.048, Supplementary Table 1) were less frequently used by 15–19-year athletes than older age groups. There was no relationship between medication and supplement use and training hours (Supplementary Table 1). Iron supplementation was more common in those athletes diagnosed with iron deficiency (*p* < 0.001).

**Table 2:** Medication and supplement use.

	***n***	**Respondents**	**Percent**
**Prescribed medication**			
Regularly use prescribed medications	98	212	46
NSAID	12	212	6
Asthma medication	12	212	6
Anti-depressant	6	212	3
Other	32	212	15
**Nutritional supplement**			
Currently using supplements	139	210	66
Not currently using supplements	71	210	34
**Current nutritional supplements**			
Protein	114	210	54
Vitamins and/or minerals	59	210	28
Caffeine	48	210	23
Probiotics	41	210	20
Oral iron	29	210	14
Fish oil	19	210	9
Creatine	14	210	7
Other	18	210	9

**Table 3:** Prevalence, indication and reported side effects of hormonal medication use.

**Medication**	***n***	**Respondents**	**Percent**
**Hormonal contraception**			
Currently using	77	207	37
Never used	76	207	37
Previously used	54	207	26
**Currently or most recently used hormonal contraception**			
COCP	79	123	64
Hormonal intrauterine device (IUD)	18	123	15
Progesterone-only pill (POP)	17	123	14
Medroxyprogesterone acetate (Depot) injection	6	123	5
Levongesterel (Jadelle) implant	3	123	2
**Combined Oral Contraceptive Pill (COCP): Reported indication**			
Contraception	73	89	82
Period manipulation	38	89	43
Enhance period regularity	31	89	35
Reduce period pain	26	89	29
Reduce acne	16	89	18
**Combined Oral Contraceptive Pill (COCP): Reported side effects**			
Nil	37	85	44
Mood disturbance	21	85	25
Weight gain	20	85	24
Light or absent periods	19	85	22
Breast tenderness	15	85	16
Heavy periods	5	85	6
Acne	4	85	5
Other	7	85	8

### Menstrual History

Twenty-two percent (45/208, data not shown) met diagnostic criteria for delayed menarche (i.e., >15 years) while 36% (74/208, data not shown) believed that their puberty onset was later than their peers. Only two respondents required intervention (medication, weight gain and/or reduction in exercise levels) to initiate menstruation.

For those not using hormonal contraception at the time of the survey (63%; 130/207, Table 3), oligomenorrhea (>3 and <9 periods in the previous year) was present in 37% (29/79) and a further 10 athletes (13%) were amenorrhoeic (<3 periods in the previous year) (data not shown). A previous diagnosis of oligo- or amenorrhea was positively associated with a history of stress fractures (*p* = 0.018, Supplementary Table 1) and disordered eating (*p* = 0.008, Supplementary Table 1) and showed a non-significant trend with iron deficiency (*p* = 0.058, Supplementary Table 1).

Thirty-seven percent (77/207) were current users of hormonal contraception (Table 3). The combined oral contraceptive pill (COCP) was most frequently utilized, primarily for contraception, but also for period manipulation, period regularity, reduction of symptoms and acne (Table 3). Fifty-six percent of COCP users reported side effects, with the most common being mood disturbance (21/85, Table 3) and weight gain (20/85, Table 3).

The majority of athletes reported menstrual cycle symptoms including pelvic pain (115/202), increased fatigue (99/202) and low back pain (94/202), disrupted sleep (58/202) that could potentially affect performance (Table 4). Menstrual cycle symptoms were less frequent in those younger (*p* = 0.022, Supplementary Table 1), non-funded (*p* < 0.001, Supplementary Table 1) or training less often (10–19 h vs. >50 h per month; *p* = 0.003, Supplementary Table 1). Dysmenorrhea (33%, 66/203), defined as the need to take pain relief with most periods, was less frequent than pelvic pain (57%, 115/202, Table 4). Thirty percent described their period nature such as to fulfill diagnostic criteria for menorrhagia (Table 4). Those with menorrhagia were more likely to have been diagnosed with iron deficiency (*p* = 0.026, Supplementary Table 1).

**Table 4:** Menstrual cycle and menstrual period characteristics.

**Menstrual cycle characteristic**	***n***	**Respondents**	**Percent**
**Regularity (when not using hormonal contraception)**			
Regular	111	206	54
Not regular	32	206	16
Don't know/Other	63	206	31
**Menstrual cycle-related symptoms**			
Pelvic pain	115	202	57
Increased fatigue	99	202	49
Low back pain	94	202	47
Disrupted sleep	58	202	29
Headaches	39	202	19
Pain in thighs	20	202	10
Nausea or vomiting	17	202	8
Other	46	202	23
Nil	41	202	20
**Requiring pain relief during menstruation**			
Never	65	203	32
Rarely	72	203	35
Most of time	54	203	27
Always	12	203	6
**Menstrual period characteristics**			
Considered heavy	60	203	30
Not considered heavy	146	203	72
Need to frequently change pads or tampons	50	201	25
Passing large blood clots	42	201	21
Flooding through protection	34	201	17
Required to use double sanitary protection	17	201	8
Struggle to complete training without changing sanitary protection	18	201	8

### Menstrual Cycle and Performance

One-third of athletes (32%; 65/203) reported their menstrual cycle was affected by training volume but no obvious trend for responses to training volumes was apparent. Outcomes associated with higher training volumes included the menstrual cycle becoming more regular (31%; 36/116), less regular (14%; 23/116), longer menses (21%; 24/116), shorter menses (17%; 20/116), and with more (30%; 35/116) or less blood loss (20%; 23/116).

Thirty-six percent (72/202) believed their menstrual cycle impacted negatively on their performance at least some or most of the time, while 28% (56/202) believed that performance was unaffected by their menstrual cycle. Four percent (8/202) believed that their menstrual cycle had a positive impact on performance at least some of the time.

The majority (86%; 174/203) reported no modification or absence from their training due to menstrual cycle symptoms. In those that modified training (*n* = 29), 90% had altered training 1–5 times in the preceding 6 months as a result of cramps or severe pain (68%), fatigue (61%), lack of motivation (54%), body aches (46%), low mood (46%), heavy bleeding (25%), gastrointestinal symptoms (21%), and/or headache (18%). One athlete reported missing a competition in the previous 4 years due to menstrual cycle-related symptoms. Fifty-four percent of athletes (112/207) tracked their menstrual cycle, using computer software (68%) or paper/electronic diary (21%).

### Appearance- and Performance Related Pressures

Seventy-three percent (141/194) believed elite sport participation was associated with pressure to have a specific physical appearance, and that such pressures may be damaging to their overall health. Fifty-four percent (109/201) believed there was pressure to conform to heteronormative notions of feminine appearance with sources of pressure identified from social media (80%; 99/124), themselves (77%; 96/124), general public (54%; 67/124), and other media (53%; 66/124). Appearance-related pressures to look a certain way (54%; 109/201) were more frequently reported than other performance-related pressures (44%; 86/196). The sources of performance-related pressure were themselves (80%; 70/87), social media (59%; 51/87) or their coach (53%; 46/87). No statistical differences were observed in the reporting of pressures based on age, performance level or training volumes.

Twenty-two athletes reported being told by their coach to lose weight for performance related reasons. Athletes reported that such comments made them feel unhappy with their body (73%; 16/22), upset (45%; 10/22), angry (32%; 7/22), demotivated (27%; 6/22), and confused (23%; 5/22). Only four (18%) athletes reported being motivated by this information.

Thirty-three (15%) reported engaging in disordered eating practices to obtain a perceived ideal body. Those practices included dietary restriction (23/219; 11%), training on rest days (16/219; 7%), purging or vomiting (4/219; 2%), or using laxatives (1/219; 0.5%).

### Communication and Sources of Information

Eighty percent (130/162) reported no barriers in communicating with support staff regarding their menstrual cycle. Of the 20% (32/162) reporting barriers, the most frequent was the gender of support staff, including male coaches (90%; 44/49), doctors (47%; 23/49), strength and conditioning coaches (43%; 21/49), and physiotherapists (24%; 12/49). Stigma of the topic (64%; 34/53), lack of support staff knowledge (51%; 27/53), and potential impact on their position (30%; 16/53) were additional factors perceived as barriers to communicating menstrual-related issues with support staff. In comparison with development athletes, a greater proportion of elite athletes reported communication barriers (*p* = 0.02, Supplementary Table 1). There was no statistically significant difference in reported barriers to communication between team and individual, or weight-bearing and non-weight bearing sports.

The most frequent source of health information was fellow athletes (40%; 75/186), general practitioner or sports doctor (37%; 69/186), their own research (30%; 55/186), coaches (23%; 42/186), sports physiotherapists (20%; 38/186), social media or websites (18%; 33/186). Examples of text provided by elite female athletes with respect to appearance and performance related pressures are provided in Table 5.

**Table 5:** Appearance and performance related pressures with examples of text provided by elite female athletes.

**Source**	**Comment**
**APPEARANCE-RELATED PRESSURES**	
Social media	*I think a lot of pressure stems from the exposure to media/social media which then cultivates as self where you are always seeing these girls (and guys) that are small and fit, lauded for being so. It can be quite hard to grasp, when you are surrounded by this…*
Self	*It's mainly me just comparing myself to others. After having to gain weight in order for me to get my period it has been mentally challenging to accept my new body and adapt to changes that have occurred*.
General public	*[I feel] pressure to appear pretty and feminine on game day, hair always done nicely*.
	*Uniforms are tiny and the pressure to look good—i.e. hair, makeup, hairless—I feel is quite big*.
	*[I feel pressure to] look girly, not look butch*.
Other media	*It is believed it is better to be lean and there is a lot of comparisons between athletes by the public and media*.
**APPEARANCE RELATED PRESSURES IN SPORT AND DAMAGE TO HEALTH**	
Sport	*The idea to look a certain way puts pressure on females around eating habits, as well as anxiety. For me I had issues with body image in the past which led to dieting and in turn, influenced injury*.
	*Some athletes have to diet very hard to make the weight which can have adverse impacts on their health, particularly when they are young and don't have enough support*.
	*We have people every day telling us how to be, what to do, how to look. This take[s] a toll on a person especially when your coach is male*.
**PERFORMANCE RELATED PRESSURES**	
Self	*Often it is promoted that the leaner you are the better you'll perform*.
	*[I feel there are pressures] to look powerful, muscular and fit*.
	*The pressure to be fit enough and have enough energy to perform is huge. However, from a performance point of view this pressure is purely related to my physical ability to complete. This pressure tends to emerge more when I feel self-conscious, or a lack of confidence in my ability*.
Social media	*For distance runners, seeing other runners in the media or on social media might make people think they need to look like them in order to be fast*.
Coach	*Coach believes being light and slim relates to how fit and fast you are. Other competitors/coach thinks you're not training if you are carrying weight*.
	*The pressures of having to make a certain weight. We are compared to each other and male coaches have been known to make comments about weights of girls weights to other girls*.
Peers	*There is the pressure of being lean and muscular, looking very toned, peers putting girls down if they are too big/ not as strong. Girls get put down if they are weaker*.
	*Indirect and direct comments about weight and appearance, snarky comments from female teammates about weight and image*.

## Discussion

This research highlighted the broad range of female medical and socio-cultural factors that have the potential to negatively influence the health and performance of elite female athletes from Aotearoa New Zealand. Our findings reiterate the need for further research specific to health and performance of female competitors, and the urgent need for strategies to address issues highlighted by these findings.

Stress fractures and concussion are sport-related injuries that carry significant morbidity and the rates we observed reflect a significant issue for elite women. The relationship between stress fractures and menstrual cycle disturbance is well recognized and reiterated by our findings (Carbon et al., 1990). Almost half those surveyed reported a diagnosis of iron deficiency or anemia, and a similar proportion reported using iron supplementation. The finding that individual sport athletes were at higher risk of illness in general, and that the risk of iron deficiency was positively associated with heavy periods and oligo-/amenorrhea illustrates need for targeted, sport-specific monitoring. While 11% of athletes reported a diagnosis of depression or anxiety, previous research on elite New Zealand athletes has suggested a rate of 21% experiencing clinically relevant symptoms of depression, suggesting a potential for under-diagnosis in elite female athletes (Beable et al., 2017).

### Menstrual Disorders and Contraceptive Choices

Approximately 8–12% of athletes had been diagnosed with either oligo/amenorrhea, RED-S or endometriosis, and slightly lower numbers were diagnosed with sexually transmitted diseases and Polycystic Ovarian Syndrome (PCOS). However, 30% reported symptoms that met criteria for menorrhagia, 33% for dysmenorrhea, and 50% for either oligo- or amenorrhea. Similarly, while only 5% reported a diagnosis of PCOS, almost one-third of athletes indicated increased training volume led to more regular menstrual cycles, consistent with PCOS. The disparity observed between diagnosed conditions and reported symptoms suggests that there may be a proportion of elite athletes with undiagnosed gynecological or hormonal pathology (Hagmar et al., 2009). Finally, noting that hormonal medication may mask underlying energy deficiency, with 50% of non-hormonally medicated athletes being oligo- or amenorrhoeic, we are concerned that 30–40% of our entire cohort (including those on hormonal contraception) could actually be in a negative energy state.

Menstrual cycle symptomatology seems individualized, with the majority of female athlete respondents reporting some symptomatology (Martin et al., 2018). Most respondents perceived their menstrual cycle to have either a negative or neutral impact on performance (Kishali et al., 2006). However, in contrast to previous literature, the majority (86%) of this cohort had not modified their training routine due to their menstrual cycle (Bruinvels et al., 2016).

Similar to previous literature, more than one-third of this elite cohort were using hormonal medication for both contraception and to manipulate their menstrual cycle (Brynhildsen et al., 1997). The COCP was the most frequently used hormonal contraception but one in four users reported weight gain and mood concerns. While basic science studies indicate a significant role of the menstrual cycle hormones on adaptation to training and performance, research data relating to performance effects of the menstrual cycle in elite athletes remains unclear (McNulty et al., 2020). Similarly, the understanding of the short and long term benefits and risks of using hormonal manipulation continues to evolve (Allaway et al., 2016). Our results suggest that reported side-effects from the use of hormonal contraceptives, irrespective of other physiological findings, may negatively affect performance in a number of athletes (Rechichi et al., 2009; Martin et al., 2018). With alternatives to the COCP increasingly available, it is important that females are fully conversant with the benefits and disadvantages of various contraceptive approaches to ensure their choices are fully informed.

These findings support the need for intensive, skilled and regular health evaluations which include specific consideration of female's health issues. Perceived negative impact of the menstrual cycle illustrates why skilled and informed conversations are required between individual athletes, their coaches and support network.

### Socio-Cultural Considerations

With pressures for body image conformity known to come from numerous sources (Haakonssen et al., 2015; Kong and Harris, 2015), this survey was designed to determine the nature of appearance- and performance-related pressures experienced across sports which have a range of cultures and ideal body types. Appearance-related pressures centered around the dual expectations of looking strong, fit and healthy, as well as pretty and feminine. Consistent with previous literature, athletes reported that pressure to look a certain way originated from both appearance- and performance-related factors, but was more frequently attributed to appearance factors (Krane et al., 2004). Strikingly, 73% of athletes felt that their sport was putting pressure on them to appear a certain way, which they believed may be damaging to their health. While it is acknowledged in the literature that elite sport may not necessarily be healthy (Chapman, 1997; Johns and Johns, 2000; Theberge, 2008; Connor, 2009), the belief by athletes that appearance-related issues were driving health concerns, mandates further investigation into the requirements and expectations of elite sport.

Mirroring research highlighting the potential impact of social media on young women's body image (Tiggerman and Slater, 2013), we observed that social media was the most recognized source of pressure for body image. Recent research has shown the benefits of specific programmes designed to support positive body image among female athletes (Voelker et al., 2019), and an evolution of attitudes toward athlete appearance, with female athletes gaining pride and confidence in strong and muscular bodies (Walters and Hefferon, 2019). This finding reiterates the importance of effective support for both athletes and support staff, and the need for further research on the role of social media and body image, disordered eating practices and related health issues in elite women's sport.

Thirty-three athletes reported engaging in disordered eating practices to obtain the “ideal” body, and twenty-two (unrelated) reported being told by their coach to lose weight for performance-related reasons. The latter approach largely resulted in negative emotional consequences for those athletes. The coach-athlete relationship is a complex power dynamic, with athletes potentially vulnerable to the advice and decisions of coaches. This is particularly applicable to male coaches with female athletes (de Haan and Norman, 2019). This finding highlights challenges within the elite athlete coaching and support staff environment, suggesting further education is required to ensure broader awareness of the consequences from instructing on weight-loss or commenting on elite female athlete bodies.

Remarkably, despite being a cohort of predominantly professional athletes with access to a range of embedded health care professionals, almost one in three reported having never received any female's health-related information. The most frequent source of health information was peers, closely followed by health professionals. While most (80%) reported no barriers to communication, barriers observed included when the coach, doctor, and other support staff were male, when there was a perceived lack of knowledge in support personnel, and the ongoing stigma related to the topic. Prior research illustrated the importance of both the gender and specific knowledge of coaches and support staff as key constraints in female athletes receiving the information and support necessary for their long-term health and wellbeing (Mukherjee et al., 2016). Given most coaches at the elite level in the New Zealand environment are male (unpublished data); communication and technical knowledge of female's health issues may be resulting in the under-reporting and management of female's health issues. Subsequently, facilitating an appropriate gender balance in coaching and support staff, as well as comprehensive education of coaches and support staff on female's health topics, must be a priority when working with elite female athletes.

## Limitations

There are several limitations of this study. As a cross sectional study, this reflects one point in time, and no causality can be inferred. The 61% response rate means it is likely that we have potentially under-reported health and sociocultural issues in elite New Zealand female athletes. The sensitive nature of the survey may mean that some athletes did not respond due to fear of anonymity or other issues, thereby biasing our results. Some sports, requiring low body mass or idealized appearance, were under-represented in this study due to low numbers of elite athletes. Aotearoa New Zealand is an increasingly multicultural society, and we acknowledge the need for more research that explores the complex relationship between culture, ethnicity and health among high performing female athletes, and particularly Māori wahine. In the context of Aotearoa, a kaupapa Māori approach to acknowledging and advancing Māori ways of knowing female athlete health is necessary, but unfortunately was beyond the scope of this survey (Thorpe et al., 2020).

A further limitation of our study is that it has not been validated by repeat responses and it is possible that the survey design led to variable response rates to some questions.

## Conclusion

This survey of elite female athletes from a diverse range of sports has highlighted the impact of a range of elements on health, wellbeing and performance. Importantly, it suggests a gap exists between rates of clinically diagnosed conditions and potentially pathological symptomatology. With one third of athletes reporting that their menstrual cycle negatively impacted on their performance, coaches and support staff require the education, skills and resources to appropriately support female athletes. However, with only half of athletes monitoring their menstrual cycles, promotion of the monitoring of menstrual cycles must be a priority.

This survey highlights the importance of the socio-cultural context in which athletes train, compete, live and socialize, on their understandings and risks of body image, and other health conditions. It was shown that most athletes report their sport exerting pressure on them to appear a certain way that might be damaging to their health, and this finding should be considered a warning for elite sport. Furthermore, the conflict invoked by appearance-related pressures in sport remain a significant burden on female athletes and must be actively addressed through education and cultural evolution. The study highlights that there are unrecognized communication barriers that may negatively influence health and performance outcomes, and it is critical that where possible these barriers are addressed through ensuring both gender diversity of coach and support staff, and comprehensive education. Finally, this survey highlighted several challenges for the organization and the clinical support of elite female sport and we hope that the findings stimulate further engagement in this important health topic.

## Summary

This manuscript reports on the findings of a survey of elite female athletes in New Zealand targeted toward a better understanding of the health concerns faced by these women. The survey highlights a prevalence of sex-specific health concerns, but also the novel observation of under-reporting and under-recognition of potentially pathological symptomatology. The study also shows a likely under-education of specific women's health related knowledge that is likely to influence the health and performance of elite female athletes. Additional to health reporting issues, the study reports that appearance-related pressures, communication challenges and social media remain major barriers facing elite female athletes. Better education of athletes, coaches and support staff is one step toward addressing these issues.

## Data Availability Statement

The original contributions presented in the study are included in the article/Supplementary Material, further inquiries can be directed to the corresponding author/s.

## Ethics Statement

The studies involving human participants were reviewed and approved by University of Waikato Human Research Ethics Committee (Health) 2018#52. The patients/participants provided their written informed consent to participate in this study.

## Author Contributions

HT, MO, SS, SB, SM, LC, and BH contributed to this manuscript by being involved in the design of the survey. All authors: analysis and interpretation of survey data, drafting and/or reviewing the work for intellectual content, and provided final approval.

## Conflict of Interest

The authors declare that the research was conducted in the absence of any commercial or financial relationships that could be construed as a potential conflict of interest.

## References

[B1] Allaway H. Mallinson R. De Souza M. (2016). Impact of combined oral contraceptive use on exercise and health in female athletes, in Exercise and Human Reproduction, eds Vaamonde D. du Plessis S. Agarwal A. (New York, NY: Springer).

[B2] Beable S. Fulcher M. Lee A. C. Hamilton B. (2017). SHARPSports mental health awareness research project: prevalence and risk factors of depressive symptoms and life stress in elite athletes. J. Sci. Med. Sport. 20, 1047–1052. 10.1016/j.jsams.2017.04.018 28601589

[B3] Bruinvels G. Burden R. Brown N. Richards T. Pedlar C. (2016). The prevalence and impact of heavy menstrual bleeding among athletes and mass start runners of the 2015 London Marathon. Br. J. Sports Med. 50:566. 10.1136/bjsports-2015-095505 26612843

[B4] Brynhildsen J. Lennartsson H. Klemetz M. Dahlquist P. Hedin B. Hammar M. (1997). Oral contraceptive use among female elite athletes and age-matched controls and its relation to low back pain. Acta Obstet. Gynecol. Scand. 76, 873–878. 10.3109/00016349709024368 9351415

[B5] Carbon R. Sambrook P. N. Deakin V. Fricker P. Eisman J. A. Kelly P.. (1990). Bone density of elite female athletes with stress fractures. Med. J. Aust. 153, 373–376. 10.5694/j.1326-5377.1990.tb125491.x 2098012

[B6] Chapman G. (1997). Making weight: lightweight rowing, technologies of power, and technologies of the self. Sociol. Sport J. 14, 205–223. 10.1123/ssj.14.3.205

[B7] Connor J. (2009). The athlete as widget: how exploitation explains elite sport. Sport Soc. 12, 1369–1377. 10.1080/17430430903204900

[B8] de Bruin A. P. Oudejans R. D. (2018). Athletes' body talk: The role of contextual body image in eating disorders as seen through the eyes of elite women athletes. J. Clin. Sports Psychol. 12, 675–698. 10.1123/jcsp.2018-0047

[B9] de Haan D. Norman L. (2019). Mind the gap: the presence of capital and power in the female athlete—male-coach relationship within elite rowing. Sports Coach. Rev. 9, 95–118. 10.1080/21640629.2019.1567160

[B10] Haakonssen E. C. Martin D. T. Jenkins D. G. Burke L. M. (2015). Race weight: perceptions of elite female road cyclists. Int. J. Sports Physiol. Perform. 10, 311–317. 10.1123/ijspp.2014-0070 25203649

[B11] Hagmar M. Berglund B. Brismar K. Hirschberg A. L. (2009). Hyperandrogenism may explain reproductive dysfunction in olympic athletes. Med. Sci. Sports Exerc. 41, 1241–1248. 10.1249/MSS.0b013e318195a21a 19461542

[B12] Hines J. C. Wendorf W. Hennen A. (2019). How do lean and non-lean female collegiate athletes view the eating disorder education they receive from their coaches? Int. J. Sports Sci. Coach. 14, 169–178. 10.1177/1747954118825060

[B13] Johns D. Johns J. (2000). Surveillance, subjectivism and technologies of power: an analysis of the discursive practice of high-performance sport. Int. Rev. Sociol. Sport 35, 219–234. 10.1177/101269000035002006

[B14] Kantanista A. Glapa A. Banio A. Firek W. Ingarden A. Malchrowicz-Mosko E.. (2018). Body image of highly trained female athletes engaged in different types of sport. Biomed. Res. Int. 2018:6835751. 10.1155/2018/6835751 29662894 PMC5831824

[B15] Kishali N. F. Imamoglu O. Katkat D. Atan T. Akyol P. (2006). Effects of menstrual cycle on sports performance. Int. J. Neurosci. 116, 1549–1563. 10.1080/00207450600675217 17145688

[B16] Kong P. Harris L. (2015). The sporting body: body image and eating disorder symptomatology among female athletes from leanness focused and nonleanness focused sports. J. Psychol. 149, 141–160. 10.1080/00223980.2013.846291 25511202

[B17] Krane V. Choi P. Y. L. Baird S. M. Aimar C. M. Kauer K. J. (2004). Living the paradox: female athletes negotiate femininity and muscularity. Sex Roles 50, 315–329. 10.1023/B:SERS.0000018888.48437.4f

[B18] Logue D. Madigan S. M. Delahunt E. Heinen M. Mc Donnell S. J. Corish C. A. (2018). Low energy availability in athletes: a review of prevalence, dietary patterns, physiological health, and sports performance. Sports Med. 48, 73–96. 10.1007/s40279-017-0790-3 28983802

[B19] Martin D. Sale C. Cooper S. B. Elliott-Sale K. J. (2018). Period prevalence and perceived side effects of hormonal contraceptive use and the menstrual cycle in elite athletes. Int. J. Sports Physiol. Perform. 13, 926–932. 10.1123/ijspp.2017-0330 29283683

[B20] McCall L. M. Ackerman K. E. (2019). Endocrine and metabolic repercussions of relative energy deficiency in sport. Curr. Opin. Endocr. Metab. Res. 9, 56–65. 10.1016/j.coemr.2019.07.005

[B21] McNulty K. L. Elliott-Sale K. J. Dolan E. Swinton P. A. Ansdell P. Goodall S.. (2020). The effects of menstrual cycle phase on exercise performance in eumenorrheic women: a systematic review and meta-analysis. Sports Med. 50, 1813–1827. 10.1007/s40279-020-01319-3 32661839 PMC7497427

[B22] Melin A. K. Heikura I. A. Tenforde A. Mountjoy M. (2019). Energy availability in athletics: health, performance, and physique. Int. J. Sport Nutr. Exerc. Metab. 29, 152–164. 10.1123/ijsnem.2018-0201 30632422

[B23] Mountjoy M. Sundgot-Borgen J. Burke L. Carter S. Constantini N. Lebrun C.. (2014). The IOC consensus statement: beyond the female athlete triad–relative energy deficiency in sport (RED-S). Br. J. Sports Med. 48, 491–497. 10.1136/bjsports-2014-093502 24620037

[B24] Mukherjee S. Chand V. Wong X. X. (2016). Perceptions, awareness and knowledge of the female athlete triad amongst coaches - are we meeting the expectations for athlete safety? Int. J. Sport Sci. Coach. 11, 545–551. 10.1177/1747954116654781

[B25] Papageorgiou M. Martin D. Colgan H. Cooper S. Greeves J. P. Y. Tang J. C.. (2018). Bone metabolic responses to low energy availability achieved by diet or exercise in active eumenorrheic women. Bone 114, 181–188. 10.1016/j.bone.2018.06.016 29933113

[B26] Rechichi C. Dawson B. Goodman C. (2009). Athletic performance and the oral contraceptive. Int. J. Sports Physiol. Perform. 4, 151–162. 10.1123/ijspp.4.2.151 19567919

[B27] Taylor S. Rampton D. (2015). Treatment of iron deficiency anemia: practical considerations. Pol. Arch. Med. Wewn. 125, 452–460. 10.20452/pamw.2888 25922941

[B28] Theberge N. (2008). ‘Just a normal bad part of what I do': elite athletes' accounts of the relationship between health and sport. Sociol. Sport J. 25, 206–222. 10.1123/ssj.25.2.206

[B29] Thorpe H. Brice J. Rolleston A. (2020). Decolonizing sport science: high performance sport, indigenous cultures, and women's rugby. Sociol. Sport J. 37, 73–84. 10.1123/ssj.2019-0098

[B30] Tiggerman M. Slater A. (2013). NetGirls: the internet, Facebook, and body image concern in adolescent girls. Int. J. Eating Disord. 46, 630–633. 10.1002/eat.22141 23712456

[B31] Voelker D. Petrie T. Huang Q. Chadran A. (2019). Bodies in motion: an empirical evaluation of a program to support positive body image in female collegiate athletes. Body Image. 28, 149–158. 10.1016/j.bodyim.2019.01.008 30716557

[B32] Walters R. Hefferon K. (2019). ‘Strength becomes her’: resistance training as a route to positive body image in women. Qualitat. Res. Sport Exerc. Health. 10.1080/2159676X.2019.1634127

