# Supplementary material for: Biological and Socio-Cultural Factors Have the Potential to Influence the Health and Performance of Elite Female Athletes: A Cross Sectional Survey of 219 Elite Female Athletes in Aotearoa New Zealand
Source: Front Sports Act Living. 2021 Feb 18;3:601420. doi: 10.3389/fspor.2021.601420 (PMC7932044; doi:10.3389/fspor.2021.601420)
Supplement: Supplementary file 1 [file Table_1.DOCX]

Table 1 Statistical evaluation of variables and health outcomes

|  |  |  | Statistics | | |
| --- | --- | --- | --- | --- | --- |
| Independent variable | **Outcome** | **Results of interest** | **χ^2^** | **P** | **Fishers p** |
| Age | Medication | Lower proportion of medication use in 15-19 year athletes | 8.43 | 0.040 | 0.036 |
|  | Supplement use | Lower proportion of supplement use in 15-19 year old athletes | 8.19 | 0.038 | 0.048 |
|  | Contraception use | Higher proportion of 15-19 yrs year old athletes don’t use contraception. Older athletes do or used to use contraception | 37.03 | <0.001 | <0.001 |
|  | Menstrual cycle symptoms | Lower proportion of 15-19 yrs year old athletes experience menstrual cycle symptoms | 9.77 | 0.020 | 0.022 |
|  | Medication use to prevent period | Higher proportion of medication use in 15-19 year old athletes | 14.71 | 0.005 | 0.001 |
|  | Appearance-related pressures | No age-related differences detected | 0.64 | 0.893 | 0.884 |
|  | Performance-related pressures | No age-related differences detected | 1.99 | 0.606 | 0.603 |
|  | Pressures causing damage to health | No age-related differences detected | 0.15 | 0.986 | 0.978 |
|  | Barriers communicating menstrual cycle issues | No age-related differences detected | 2.49 | 0.488 | 0.536 |
| Funding (fully funded (carded) versus development level athlete) | Supplement use | Higher proportion of carded athletes take supplements | 14.48 | <0.001 | <0.001 |
|  | Medication |  | 1.91 | 0.379 | 0.399 |
|  | Injury | Non-carded and P2P athletes have a higher proportion of injuries | 12.24 | 0.002 | 0.002 |
|  | Iron deficiency |  | 3.20 | 0.204 | 0.196 |
|  | Iron supplement use | Higher proportion of supplement use in carded than non-carded | 7.24 | 0.025 | 0.024 |
|  | Late puberty onset |  | 0.30 | 0.894 | 0.897 |
|  | Illness |  | 3.76 | 0.148 | 0.153 |
|  | Contraception use | Higher proportion of carded athletes using contraception | 15.95 | 0.004 | 0.003 |
|  | Menstrual cycle symptoms | Higher proportion of symptoms in carded athletes | 15.15 | <0.001 | <0.001 |
|  | Appearance-related pressures | No funding status related differences detected | 1.24 | 0.583 | 0.555 |
|  | Performance-related pressures | No funding status related differences detected | 0.02 | 1.00 | 1.00 |
|  | Pressures causing damage to health | No funding status related differences detected | 1.83 | 0.430 | 0.400 |
|  | Barriers communicating menstrual cycle issues | Higher proportion of barriers communicating menstrual cycle issues in carded fully funded athletes | 8.64 | 0.015 | 0.020 |
| Training hours | Injury | No relationship with training hours detected | 2.39 | 0.513 | 0.509 |
|  | Late puberty onset | No relationship with training hours detected | 2.55 | 0.459 | 0.469 |
|  | Menstrual cycle symptoms | Lower proportion of symptoms in athletes with fewer training hours | 13.35 | 0.004 | 0.003 |
|  | Training disruption due to period | No relationship with training hours detected | 1.64 | 0.669 | 0.628 |
|  | Medication use to prevent period | No relationship with training hours detected | 2.26 | 0.518 | 0.542 |
|  | Appearance-related pressures | No relationship with training hours detected | 1.78 | 0.622 | 0.639 |
|  | Performance-related pressures | No relationship with training hours detected | 1.88 | 0.608 | 0.617 |
|  | Pressures causing damage to health | No relationship with training hours detected | 0.53 | 0.903 | 0.893 |
|  | Barriers communicating menstrual cycle issues | No relationship with training hours detected | 3.46 | 0.352 | 0.366 |
|  |  |  |  |  |  |
|  |  |  |  |  |  |

|  |  |  | **Statistics** | | |
| --- | --- | --- | --- | --- | --- |
| **Independent variable** | **Outcome** | **Results of interest** | **χ^2^** | **P** | **Fishers p** |
| **Team vs. individual sports** | Injury | *Non-significant trend towards a higher proportion of injuries in athletes from team sports* | 5.84 | 0.059 | 0.053 |
|  | Mental health issues | No relationship with team or individual sport | 4.23 | 0.122 | 0.111 |
|  | Illness | A higher proportion of individual sports athletes have been diagnosed with an illness | 6.23 | 0.047 | 0.048 |
|  | Menstrual cycle symptoms | No relationship with team or individual sport | 1.53 | 0.477 | 0.491 |
|  | Training hours | A higher proportion of athletes from individual sports have a higher training load | 47.4 | <0.001 | <0.001 |
|  |  |  |  |  |  |
|  | Barriers communicating menstrual cycle issues | No relationship with team or individual sport | 3.24 | 0.194 | 0.181 |
| **Weightbearing vs. non-weightbearing sports** | Injury | A higher proportion of injuries observed in athletes who compete in weightbearing sports | 7.29 | 0.028 | 0.023 |
|  | Mental health issues | Athletes in non-weight bearing sports have a higher proportion of mental health issues | 8.12 | 0.024 | 0.014 |
|  | Illness | No relationship observed with weightbearing or non-weightbearing sports | 1.16 | 0.615 | 0.579 |
|  | Menstrual cycle symptoms | No relationship observed with weightbearing or non-weightbearing sports | 1.43 | 0.524 | 0.544 |
|  | Training hours | A higher proportion of weightbearing sport athletes had shorter training hours per week | 44.9 | <0.001 | <0.001 |
|  | Barriers communicating menstrual cycle issues | No relationship observed with weightbearing or non-weightbearing sports | 3.78 | 0.170 | 0.125 |
| **Iron deficiency** |  |  |  |  |  |
|  | Illness | No relationship with iron deficiency | 1.14 | 0.292 | 0.292 |
|  | Heavy periods | A higher proportion of athletes with heavy periods are iron deficient | 5.76 | 0.026 | 0.021 |
|  | Use of NSAIDS | No relationship with iron deficiency | 0.69 | 0.553 | 0.554 |
|  | Menstrual cycle symptoms | No relationship with iron deficiency | 0.17 | 0.751 | 0.750 |
|  | Oligo/amenorrhea | *Non significant trend towards a higher proportion of athletes with oligo/amenorrhea having an iron deficiency* | 4.16 | 0.054 | 0.058 |
|  | Disordered eating | No relationship with iron deficiency | 0.83 | 0.472 | 0.478 |
|  |  |  |  |  |  |
| **Stress fractures** | Training hours | No relationship with stress fractures | 8.19 | 0.074 | 0.101 |
|  |  |  |  |  |  |
|  | Oligo/Amenorrhea | A higher proportion of athletes with oligo/amenorrhea have had stress fractures | 6.44 | 0.018 | 0.022 |
| **Oligo/amenorrhea** | Age | No relationship with Oligo/Amenorrhea | 1.82 | 0.623 | 0.711 |
|  | Age of menarche | No relationship with Oligo/Amenorrhea | 2.97 | 0.130 | 0.128 |
|  | Training hours | No relationship with Oligo/Amenorrhea | 3.06 | 0.557 | 0.664 |
|  | Disordered eating | A higher proportion of athletes with oligo/amenorrhea have been diagnosed with an eating disorder | 11.61 | 0.009 | 0.008 |
