# Supplementary material for: Biological and Socio-Cultural Factors Have the Potential to Influence the Health and Performance of Elite Female Athletes: A Cross Sectional Survey of 219 Elite Female Athletes in Aotearoa New Zealand
Source: Front Sports Act Living. 2021 Feb 18;3:601420. doi: 10.3389/fspor.2021.601420 (PMC7932044; doi:10.3389/fspor.2021.601420)
Supplement: Supplementary file 2 [file Presentation_1.PDF]

ATHLETE WINS SURVEY - WHISPA

CONSENT

There is no obligation for you to be involved in this study. If you do not participate your normal health management and training/competition plan will be followed. If you decide to participate in the study and later feel that you no longer wish to be a part of it, you may withdraw from the study at any time without prejudice to any current or future medical treatment.

Your information relating to this study and any other information received will be kept strictly confidential. Your identity will not be revealed and your confidentiality will be protected in any reviews and reports of this study which may be published or shared internally with NSO's.

As this study involves an online survey, it is not anticipated that you will suffer any adverse effects of participating. However, if you have any concerns regarding outcomes from your participation, please contact one of the principal researchers: Dr. Stacy Sims, ssims@waikato.ac.nz; AP Holly Thorpe, holly.thorpe@waikato.ac.nz; and/or Dr. Bruce Hamilton, bruce.hamilton@hpsnz.org.nz.

By continuing you consent to the above. Thank you.

ATHLETE WINS SURVEY - WHISPA

1. Demographic Information

1. How old are you? (years)

2. In which sport are you an athlete?

3. Discipline (where appropriate)

4. Please tick which category best describes your level of funding support

- Pathway to Podium athlete (P2P)
- Carded supported
- Non carded supported

5. Which ethnicity do you mainly identify with?

- Maori
- NZ European
- Pacific Island
- Asian
- Middle Eastern
- Latin American
- African
- Other
- Other (please specify)

6. How many years have you played competitive sport?

7. Approximately how many hours have you trained and/or competed over the last month ?

HIGH PERFORMANCE SPORT NEW ZEALAND

ATHLETE WINS SURVEY - WHISPA

2. General Medical Information

These questions relate to your general medical history. If you have any concerns regarding the information included below, please discuss with the medical director for your sport.

8. Do you currently take any regular prescribed medications (pills, lotions, injections)?

HIGH PERFORMANCE SPORT NEW ZEALAND

ATHLETE WINS SURVEY - WHISPA

9. Please specify what prescribed medications you take on a regular basis

- [ ] Non steroid anti-inflammatory
- [ ] Asthma medication
- [ ] Oral contraceptive
- [ ] Thyroid medication
- [ ] Anti-depressant
- [ ] Other (please specify)

10. Do you currently take any supplements?

HIGH PERFORMANCE SPORT NEW ZEALAND

ATHLETE WINS SURVEY - WHISPA

11. Please tick all supplements you are currently taking.

- [ ] Creatine
- [ ] Caffeine
- [ ] Protein
- [ ] Fish oil
- [ ] Vitamins and/or minerals (including calcium/vitamin D)
- [ ] Beta alanine
- [ ] Oral iron supplementation
- [ ] Glucosamine
- [ ] Probiotics
- [ ] Other (please specify)

12. Have you ever been diagnosed with any of the following? (tick all that apply)

- [ ] Stress fracture(s)
- [ ] Concussion
- [ ] Disordered eating
- [ ] Depression / Anxiety
- [ ] Asthma
- [ ] Haemochromatosis (iron overload disorder)
- [ ] Low bone density
- [ ] Hypothyroidism (low thyroid activity)
- [ ] ACL rupture
- [ ] Oligo/amenorrhoea (reduced/no periods)

13. Have you ever been diagnosed with iron deficiency and/or anaemia?

- Yes
- No

14. Have you ever used oral iron supplementation or received an iron injection or infusion?

- Yes
- No

15. Have you ever had surgery for gynaecological/women's health related issues?

- Yes
- No

HIGH PERFORMANCE SPORT NEW ZEALAND

ATHLETE WINS SURVEY - WHISPA

16. What was the surgery for?

HIGH PERFORMANCE SPORT NEW ZEALAND

ATHLETE WINS SURVEY - WHISPA

3. Gynaecological Information

These questions relate to your personal gynaecological history. If you have any concerns about any of the below we advise discussing this with the medical director for your sport.

17. At what age did you have your first period?

18. Did your first period occur naturally? (i.e. without medical intervention)

19. What kind of treatment was used to start your menstrual cycle? (tick all that apply)

- [ ] Hormonal treatment
- [ ] Weight gain
- [ ] Other (please specify)
- [ ] Reduced amount of exercise
- [ ] Increased nutritional intake

20. Do you think that puberty was later for you than for your friends?

21. Has a doctor ever told you that you might have any of the following conditions? (tick all those that apply)

- [ ] Polycystic ovary syndrome
- [ ] Endometriosis
- [ ] Premature ovarian failure (early menopause)
- [ ] Excess of the hormone prolactin
- [ ] Female athlete triad or relative energy deficiency in sport (RED-S)
- [ ] Sexually transmitted infection
- [ ] None of the above

Any other gynaecological diagnosis? Please specify.

22. Do you have any health concerns related to the following? (tick all that apply)

- [ ] Acne
- [ ] Unwanted hair-growth on your face, abdomen or thighs
- [ ] Milky discharge from the your breasts
- [ ] None of the above

23. If you answered yes to 'milky discharge from your breasts' we recommend you make an appointment with your general practitioner or medical director for your sport.

ATHLETE WINS SURVEY - WHISPA

4. Hormonal Contraception

This section relates to questions about hormonal contraception. Hormonal contraception may include the oral contraceptive pill, the progesterone only pill/mini pill, the Mirena intrauterine device, the Jadelle implant and the Depo Provera injection.
It does not include barrier contraception such as condoms.

24. Have you ever used hormonal contraception (for any reason)?

- Yes, and **currently** using hormonal contraception
- Yes, but **no longer** using hormonal contraception
- No, I have never used hormonal contraception

ATHLETE WINS SURVEY - WHISPA

25. Which hormonal contraception are you currently using, or have you most recently used?

Combined oral contraceptive pill

26. Why did you use the combined oral contraceptive pill? Please tick all that apply.
(examples of combined oral contraceptive pill- Ava, Loette, Levlen, Microgynon, Microlut, Norimin, Yasmin or Yaz, Ginet or Estelle, Monofeme, Mercilon)

- [ ] To make my periods regular
- [ ] To reduce period pain
- [ ] To try and skip my period
- [ ] Contraception
- [ ] Other (please specify)
- [ ] To reduce acne
- [ ] Unsure; I was prescribed it by my doctor
- [ ] To prevent injury

27. How long did you or have you used it for?

- ○ less than 3 months
- ○ 3-6 months
- ○ More than 6 months

28. Did you or have you noticed any side effects from this combined oral contraceptive pill?

- [ ] Weight gain
- [ ] Weight loss
- [ ] Heavy periods
- [ ] Light or no periods
- [ ] Migraines
- [ ] Performance deficit
- [ ] Performance enhancement
- [ ] Mood disturbance
- [ ] Breast tenderness
- [ ] Acne
- [ ] No side effects

Progesterone only pill ('mini-pill')

29. Why did you use the Progesterone only pill / "mini-pill" (eg: Cerazette, Noriday)? (tick all that apply)

- [ ] To make my periods regular
- [ ] To reduce acne
- [ ] To reduce period pain
- [ ] Unsure; I was prescribed it by my doctor
- [ ] To try and skip my period
- [ ] To prevent injury
- [ ] Contraception
- [ ] Other (please specify)

30. How long did you or have you used it for?

- ( ) less than 3 months
- ( ) 3-6 months
- ( ) more than 6 months

31. Did you or have you noticed any side effects on the Progesterone only (mini) pill? (please tick all that apply)

- [ ] Weight gain
- [ ] Breast tenderness
- [ ] Weight loss
- [ ] Acne
- [ ] Heavy periods
- [ ] Performance deficit
- [ ] Light or no periods
- [ ] Performance enhancement
- [ ] Migraines
- [ ] No side effects
- [ ] Mood disturbances

Hormonal intrauterine contraceptive device (eg: Mirena IUD, Jaydess IUD)

32. Why did you use this hormonal intrauterine device (IUD)? (please check all that apply)

- [ ] To make my periods regular
- [ ] To try and skip my period
- [ ] To reduce period pain
- [ ] Contraception
- [ ] Other (please specify)
- [ ] To reduce acne
- [ ] To prevent injury
- [ ] Unsure; I was prescribed it by my doctor

33. How long did you or have you used it for?

- ( ) less than 3 months
- ( ) 3-6 months
- ( ) more than 6 months

34. Did you or have you noticed any side effects using the hormonal IUD? (please check all that apply)

- [ ] Weight gain
- [ ] Weight loss
- [ ] Heavy periods
- [ ] Light or no periods
- [ ] Mood disturbances
- [ ] Breast tenderness
- [ ] Acne
- [ ] Performance deficit
- [ ] Performance enhancement
- [ ] No side effects

Depot injection.

35. Why did you use the Depot injection?

- [ ] To make my periods regular
- [ ] To try and skip my periods
- [ ] To reduce period pain
- [ ] Contraception
- [ ] Other (please specify)
- [ ] To reduce acne
- [ ] To prevent injury
- [ ] Unsure; I was prescribed it by my doctor

36. How long did you, or have you used it for?

- [ ] less than 3 months
- [ ] 3-6 months
- [ ] more than 6 months

37. Did you/have you noticed any side effects using the Depo Injection?

- [ ] Weight gain
- [ ] Weight loss
- [ ] Heavy periods
- [ ] Light or no periods
- [ ] Mood disturbance
- [ ] Breast tenderness
- [ ] Acne
- [ ] Performance enhancement
- [ ] Performance deficit
- [ ] No side effects

Jadelle implant

38. Why did you use this Jadelle implant? (small implant under the skin)

- [ ] To make my periods regular
- [ ] To reduce period pain
- [ ] To try and skip my period
- [ ] Contraception
- [ ] To improve acne
- [ ] To prevent injury
- [ ] Unsure; I was prescribed it by my doctor
- [ ] Other (please specify)

39. How long did you, or have you used it for?

- ( ) less than 3 months
- ( ) 3-6 months
- ( ) more than 6 months
- ( ) Other (please specify)

40. What side effects did you experience with the Jadelle implant?

- [ ] Weight gain
- [ ] Weight loss
- [ ] Heavy periods
- [ ] Light or no periods
- [ ] Mood disturbance
- [ ] Breast tenderness
- [ ] Acne
- [ ] Performance enhancement
- [ ] Performance deficit
- [ ] No side effects

Menstrual Cycle

These questions relate to your menstrual cycle.

41. How many periods have you had in the last twelve months?

42. Do you track your own menstrual cycle?

- Yes
- No

HIGH PERFORMANCE SPORT NEW ZEALAND

ATHLETE WINS SURVEY - WHISPA

43. How do you record your menstrual cycle?

- Training Software
- Smart phone App
- Paper or electronic diary
- Other (please specify)

44. How useful do you find your method for tracking your cycle?

Not useful at all — Extremely useful

45. When you don't use hormonal contraception are your periods regular? (21 -35 days)

- Yes
- No
- Don't know
- I have never used a form of hormonal contraception

46. Relating to your menstrual cycle do you experience any of the following symptoms? (Please check all that apply)

- [ ] Pelvic pain (pain in the lower part of your belly)
- [ ] Pain when opening your bowels
- [ ] Bleeding when opening your bowels
- [ ] Pain on passing urine
- [ ] Blood in your urine
- [ ] Low back pain
- [ ] Deep pelvic pain during sex
- [ ] Pain in upper legs or thighs
- [ ] Nausea or vomiting
- [ ] Increased fatigue
- [ ] Disrupted sleep
- [ ] Headaches
- [ ] None of these

Other (please specify)

47. Do you take pain relief during your period?

48. Do you consider your periods to be heavy?

- [ ] Yes
- [ ] No

49. During your period do you regularly (tick all that apply)

- [ ] Pass large blood clots
- [ ] Flood through your protection to clothes or bedding
- [ ] Needing to very frequently change sanitary pads or tampons
- [ ] Needing to wear double sanitary protection
- [ ] Struggle to complete training session without changing protection
- [ ] None of these

50. When your training volume, intensity or duration changes do you experience changes in your menstrual cycle?

- ( ) Yes
- ( ) No
- ( ) Don't know

51. What specific changes in your menstrual cycle do you notice when you reduce training volume, intensity or duration?

- [ ] I bleed less
- [ ] I bleed more
- [ ] I bleed fewer days
- [ ] I bleed more days
- [ ] My bleeding stops
- [ ] My monthly cycle is more regular
- [ ] My monthly cycle is less regular
- [ ] Other (please specify)

52. In the last six months have you missed or modified any ***training*** due to menstrual cycle related symptoms?

- [ ] Yes
- [ ] No

HIGH PERFORMANCE SPORT NEW ZEALAND

ATHLETE WINS SURVEY - WHISPA

53. How many times in the last six months have you had to miss or modify a training session due to menstrual cycle related symptoms?

54. Relating to your menstrual cycle what symptoms have caused you to miss or modify training? (please tick all that apply)

- [ ] Heavy bleeding
- [ ] Severe pain or cramps
- [ ] Fatigue
- [ ] Nausea or vomiting
- [ ] Diarrhoea or constipation
- [ ] Body aches
- [ ] Low mood
- [ ] Lack of motivation
- [ ] Headache
- [ ] Other (please specify)

55. In the last four years (Olympic cycle) have you missed a ***competition*** due to menstrual cycle related symptoms?

- [ ] Yes
- [ ] No

56. What menstrual cycle related symptoms have prevented you from competing? (please tick all that apply)

- [ ] Heavy bleeding
- [ ] Severe period pain or cramping
- [ ] Fatigue
- [ ] Nausea or vomiting
- [ ] Diarrhoea or constipation
- [ ] Other (please specify)
- [ ] Body aches
- [ ] Low mood
- [ ] Lack of motivation
- [ ] Headaches

57. In the last four years do you believe menstrual cycle related symptoms have impacted your performance? (tick all that apply)

- [ ] Yes, I go better when I have my period
- [ ] Yes, I go worse when I have my period
- [ ] Yes, I got better when I am ovulating
- [ ] Yes, I go worse when I am ovulating
- [ ] I am not sure
- [ ] No, it doesn't seem to affect my performance

58. Do you, or have you, used medication to prevent menstruation during a competition?

- [ ] Yes
- [ ] No

HIGH PERFORMANCE SPORT NEW ZEALAND
ATHLETE WINS SURVEY - WHISPA

59. What medication have you used?

60. Which of the following best reflects *your beliefs* about having a period whilst competing?

HIGH PERFORMANCE SPORT NEW ZEALAND

ATHLETE WINS SURVEY - WHISPA

6. PRESSURE IN SPORT

This section relates to particular concerns relating to pressure that may affect women in elite sport.

61. Do you feel there are ***appearance-related*** pressures to look a particular way in your sport?

- [ ] Yes
- [ ] No
- [ ] Not sure

HIGH PERFORMANCE SPORT NEW ZEALAND

ATHLETE WINS SURVEY - WHISPA

62. What are the sources of this pressure? (please check all that apply)

- [ ] Self
- [ ] Coach
- [ ] Peers
- [ ] Parents
- [ ] Media
- [ ] Social media
- [ ] Sports Organisation
- [ ] Public
- [ ] Support staff
- [ ] Other

63. Can you please explain what these pressures are?

64. Do you feel there are ***performance-related*** pressures to look a particular way in your sport?

- [ ] Yes
- [ ] No
- [ ] Not sure

HIGH PERFORMANCE SPORT NEW ZEALAND

ATHLETE WINS SURVEY - WHISPA

65. What are the sources of the pressures? (please check all that apply)

- [ ] Self
- [ ] Coach
- [ ] Peers
- [ ] Parents
- [ ] Media
- [ ] Social media
- [ ] Sports organisaton
- [ ] Public
- [ ] Support Staff
- [ ] Other

66. Can you please explain what these pressures are?

67. Do you feel conflict between the ideal body for performance in your sport (e.g. muscular, being lean) and broader concepts around femininity?

- [ ] Yes
- [ ] No
- [ ] Not sure

68. Please explain more about this conflict you experience.

69. Do you feel the culture of your sport in ***New Zealand*** puts pressure on female athletes to look a particular way that may be damaging to women's health?

- [ ] Yes
- [ ] No

ATHLETE WINS SURVEY - WHISPA

70. Please comment on how the culture of your sport in ***New Zealand*** puts pressure on female athletes to look a particular way that may be damaging to women's health?

71. Have you used any of the following to try to obtain the "ideal" body for performance or appearance reasons? (please tick all that apply)

- [ ] Dieting or very restrictive nutrition practices
- [ ] Vomiting or purging
- [ ] Training on rest days
- [ ] Use of medication or supplements
- [ ] Use of laxatives
- [ ] Other (please specify)

72. Do you discuss menstrual cycle related issues with your coach?

◯ Yes

◯ No

73. Has a coach (past or present) ever encouraged you to lose weight for performance reasons?

◯ Yes

◯ No

74. If yes, how did this comment make you feel? (please tick all that apply)

- [ ] Motivated
- [ ] Demotivated
- [ ] Confused
- [ ] Upset
- [ ] Other (please specify)
- [ ] Angry
- [ ] Unhappy with your body
- [ ] No emotional response

75. Do you experience any barriers communicating menstrual-related issues with support staff?

◯ Yes

◯ No

HIGH PERFORMANCE SPORT NEW ZEALAND

ATHLETE WINS SURVEY - WHISPA

76. Specifically do you experience barriers with (tick all that apply)

- [ ] Female coach
- [ ] Male coach
- [ ] Female sports scientist
- [ ] Male sports scientist
- [ ] Female nutritionist
- [ ] Male nutritionist
- [ ] Female doctor
- [ ] Male doctor
- [ ] Female strength and conditioner
- [ ] Male strength and conditioner
- [ ] Male physiotherapist
- [ ] Female physiotherapist

77. What do you perceive are the barriers?

- [ ] Gender of staff
- [ ] Lack of staff knowledge of these issues
- [ ] Stigma of this topic
- [ ] Concerns about the nature of the conversation impacting your position in sport/team (e.g. deselection from competition)
- [ ] Cultural differences between yourself and staff
- [ ] Other

Other (please specify)

HIGH PERFORMANCE SPORT NEW ZEALAND

ATHLETE WINS SURVEY - WHISPA

78. If any, where have you received information regarding women's health in high level sport? (please tick all that apply)

- [ ] I haven't received any information
- [ ] Coach
- [ ] National Sporting Organisation
- [ ] Medical Director for my sport
- [ ] General Practitioner/Sports Doctor
- [ ] Endocrinologist/Gynaecologist
- [ ] Books
- [ ] Other (please specify)
- [ ] Fellow athletes
- [ ] Websites
- [ ] Social Media
- [ ] Sports Physician
- [ ] Sports physiotherapist
- [ ] My own research

79. How useful do you feel this information has been for you?

Very unhelpful (inaccurate or contradictory information) — Very helpful

HIGH PERFORMANCE SPORT NEW ZEALAND

ATHLETE WINS SURVEY - WHISPA

THANK YOU FOR COMPLETING AND PARTICIPATING IN THIS SURVEY

We are really grateful to you for completing this survey.

If you have any concerns, questions, or feel you need to speak to someone please contact one of the investigators of this survey, your National Sporting Organisation Medical Director, or your General Practitioner.
